# Role of Surfactant Micellization for Enhanced Dissolution of Poorly Water-Soluble Cilostazol Using Poloxamer 407-Based Solid Dispersion via the Anti-Solvent Method

**DOI:** 10.3390/pharmaceutics13050662

**Published:** 2021-05-05

**Authors:** Gang Jin, Hai V. Ngo, Jing-Hao Cui, Jie Wang, Chulhun Park, Beom-Jin Lee

**Affiliations:** 1 College of Pharmacy, Ajou University, Suwon 16499, Korea; jg@jlict.edu.cn (G.J.); nvhai@ajou.ac.kr (H.V.N.); chulhun@ualberta.ca (C.P.); 2 School of Chemical and Pharmaceutical Engineering, Jilin Institute of Chemical Technology, Jilin 132022, China; 3 College of Pharmaceutical Science, Soochow University, Suzhou 215123, China; jhcui@suda.edu.cn; 4 Students Innovation and Entrepreneurship Center, Jilin Institute of Chemical Technology, Jilin 132022, China; wj@jlict.edu.cn; 5 Faculty of Pharmacy and Pharmaceutical Sciences, University of Alberta, Edmonton, AB T6G 2H7, Canada; 6 Institute of Pharmaceutical Science and Technology, Ajou University, Suwon 16499, Korea

**Keywords:** water-insoluble cilostazol, solid dispersion, anti-solvent method, micellization of sodium lauryl sulfate, enhanced dissolution, partial amorphous state, molecular interaction, particle size, contact angle

## Abstract

This study aimed to investigate the role of micellization of sodium lauryl sulfate (SLS) in poloxamer 407 (POX)-based solid dispersions (POX-based SDs) using the anti-solvent method in enhancing the dissolution rate of practically water-insoluble cilostazol (CLT). Herein, SLS was incorporated into CLT-loaded SDs, at a weight ratio of 50:50:10 of CLT, POX, and SLS by three different methods: anti-solvent, fusion (60 °C), and solvent (ethanol) evaporation. The SDs containing micellar SLS in the anti-solvent method were superior in the transformation of the crystalline form of the drug into a partial amorphous state. It was notable that there was an existence of a hydrophobic interaction between the surfactant and the hydrophobic regions of polymer chain via non-covalent bonding and the adsorption of micellar SLS to the POX-based SDs matrix. Moreover, SLS micellization via the anti-solvent method was effectively interleaved in SDs and adhered by the dissolved CLT, which precluded drug particles from aggregation and recrystallization, resulting in improved SD wettability (lower contact angle) and reduced particle size and dissolution rate. In contrast, SDs without micellar SLS prepared by the solvent method exerted drug recrystallization and an increase of particle size, resulting in decreased dissolution. Incorporation of surfactant below or above critical micellar concentration (CMC) in SDs using the anti-solvent method should be considered in advance. Dissolution results showed that the pre-added incorporation of micellar SLS into POX-based SDs using the anti-solvent method could provide a way of a solubilization mechanism to enhance the dissolution rate of poorly water-soluble drugs.

## 1. Introduction

With the discovery of a significant number of poorly soluble compounds, formulation technologies to overcome solubility and limited bioavailability have played an increasingly important role in the formulation of oral solid drug products [1,2]. Solid dispersion (SD) technology has been widely used to encapsulate hydrophobic drugs into a hydrophilic matrix and involves the transformation of crystalline drugs into amorphous forms through the interaction between drugs and carriers to improve drug dissolution [3,4,5,6,7]. Although amorphous SDs could be beneficial in enhancing the dissolution rate of the drugs, the supersaturated state of the dissolved drugs in SDs might lead to drug precipitation, consequently reducing drug bioavailability. This issue can be tackled by the addition of surface-active agents (SAAs), also known as surfactants, to SDs as carriers or additives to further improve drug dissolution performance, reduce recrystallization, and improve physical stability [8,9,10]. Moreover, SAAs with amphiphilic structures can enhance the wettability of SDs by adsorbing onto the surface of drug particles or self-assembling to encapsulate the hydrophobic drugs for enhancing dissolution of poorly water-soluble drugs. However, the roles of micellization of SAA in SDs on the dissolution behaviors of poorly water-soluble drugs remains questionable. Micellization is a dynamic phenomenon in which monomeric surfactant molecules associate to form a micelle above the critical micelle concentration (CMC) in the solution at a given temperature.

Among the several SAAs investigated, anionic surfactants such as sodium lauryl sulfate (SLS) have been widely used for enhancing the dissolution of various poorly water-soluble drugs [11,12,13]. SLS is a widely used anionic surfactant for in vitro dissolution testing, owing to its similarity with bio-relevant mediums [14,15,16]. Furthermore, SLS is often used as a solubilizer to improve the aqueous solubility and reduce the precipitation of poorly water-soluble drugs to a certain extent by preserving a high drug concentration in the aqueous medium during the dissolution process in dosage forms [17,18,19,20,21,22]. However, the way of incorporation of SLS in SD for effective improvement of dissolution and a safety issue with SLS in the preparation of SD are very crucial. Therefore, the pre-added incorporation methods of surfactants in the concentration below or above the critical micellar concentration (CMC) in the SD preparation process should be carefully considered. However, the role of pre-added micellar SLS in SDs has rarely been investigated according to the different preparation methods, although the incorporation of SLS has been studied in various SDs containing different therapeutic agents. There have been limited studies about the effect of these surfactant-polymer complexes on drug solubilization, especially the role of micellar surfactant in SDs following this concept [23].

In this study, we hypothesized that the incorporation of micellar SLS would be beneficial to interleave SLS inside SDs, promoting an adsorption between micellar SLS and POX-based SDs and exerting a stabilization effect on drug release. Thereby, we focused on discovering the role of micellar SLS in SDs via an anti-solvent method as compared to other methods, such as the fusion method and solvent evaporation method. The dissolution-enhancing mechanism was elucidated in terms of solubility, dissolution, particle size, and wettability (contact angle). The molecular interaction and drug crystallinity in SDs were also investigated using diverse instrumental analysis. Cilostazol (CLT) was chosen as a model drug. CLT has been widely used for the treatment of cardiovascular diseases as it can control the aggregation of blood platelets and promote vascular relaxation by inhibiting phosphodiesterase types [24]. However, CLT is a weakly basic drug (pKa = 11.8), extremely hydrophobic (6 µg/mL at 37 °C in aqueous solution), and is classified as a BCS II drug. Meanwhile, several approaches have been used to enhance the solubility and dissolution rate of CLT [25,26,27,28,29,30,31].

## 2. Materials and Methods

### 2.1. Materials

CLT was obtained from Chemagis Co. Ltd. (Ramat Hovav, Israel). SLS was purchased from Sigma-Aldrich (Saint-Louis, MO, USA). Hydroxypropyl methylcellulose (HPMC.6 cps) was obtained through the courtesy of Richwood Co. (Seoul, Korea). Poloxamer 407, polyvinylpyrrolidone K30 (PVP K30), D-α-tocopherol polyethylene glycol 1000 succinate (TPGS), and soluplus were supplied by BASF Chemicals (Ludwigshafen, Germany). Polyethylene glycol 6000 (PEG 6000) was obtained from Yakuri Pure Chemicals (Tokyo, Japan). All the other chemicals were of analytical grade and were used without further purification.

### 2.2. Preparation of CLT-SDs

#### 2.2.1. Solvent Evaporation Method

First, 500 mg (10 doses) of CLT was dissolved completely in 50 mL of absolute ethanol under magnetic stirring (controlled at 45 °C) to form a transparent solution. Then, 500 mg of POX and 100 mg of SLS were added to this solution, and stirring continued for 2 h. The mixture was then placed in an oven, maintained at 50 °C for solvent evaporation. All the drugs and excipients were pulverized thoroughly using a pestle and mortar and passed through a 40-mesh sieve before and after preparation.

#### 2.2.2. Fusion Method

POX (500 mg) was heated to a molten state at 60 °C, and 500 mg of CLT and 100 mg of SLS, which were sieved, were added to it. The mixture was stirred continuously until it was uniform and then quickly placed in the deep freezer (−20 °C) for pre-freezing. The congealed mass was then pulverized thoroughly using a pestle and mortar and passed through a 40-mesh sieve for further use.

#### 2.2.3. Anti-Solvent Method

To obtain the uniformity of particle size and rapid dispersion in the solution, CLT (500 mg) and POX (500 mg) were ground and sieved and then completely dissolved in 50 mL of absolute ethanol at 45 °C. Simultaneously, 100 mg of SLS was dissolved in 30 mL (above CMC of SLS) or 500 mL (below CMC of SLS) of deionized water or enzyme-free gastric fluid (pH 1.2). Next, the CLT solution was quickly added to the SLS aqueous solution under continuous stirring for 3 min, followed by sonication for 30 min to disperse the drug particles homogeneously in the SLS solution. The resulting cosolvent was removed by rotary evaporation, and the remaining solution was then freeze-dried for 24 h at −83 °C to obtain the solid powders. For the purpose of comparison, each component was accurately weighed in a specific ratio, placed in a test tube with a cap, and vortexed for 10 min to obtain the physical mixture (PM). The formulation compositions used to prepare CLT-SDs and PM are shown in Table 1.

### 2.3. Solubility Study

Deionized water, enzyme-free gastric (pH 1.2) and intestinal fluid (pH 6.8), and 1% (*w*/*v*) aqueous solutions of various excipients, as shown in Table 2, were used as media for solubility-testing experiments. An excess amount of CLT was added to the tube containing 10 mL of media and shaken in a water bath, maintained at 37 °C, at 100 rpm for 24 h. Next, the samples were centrifuged and immediately diluted with the mobile phase for analysis using high-performance liquid chromatography (HPLC). Enzyme-free gastric fluid was prepared by dissolving sodium chloride (NaCl) in deionized water, and the pH was adjusted with 7.4% diluted HCl solution. Enzyme-free intestinal fluid (pH 6.8) was prepared by dissolving monobasic potassium phosphate (KH_2_PO_4_) in deionized water and then adding 1 N NaOH solution to adjust the pH to 6.8.

### 2.4. In Vitro Dissolution Studies

Dissolution rate studies were performed in 900 mL of enzyme-free gastric fluid (pH 1.2) as dissolution media, containing different concentrations of SLS (0.1%, 0.3%, and 0.5% *w*/*v*) or no SLS, at 37 ± 0.5 °C at a rotation speed of 50 rpm, using the USP dissolution II paddle method. The SD systems equivalent to 50 mg of CLT were exposed to dissolution media in powder form. The powdered sample was not floating and sufficiently wet because the formulation contained a certain amount of surfactant. At 5, 10, 15, 30, 45, 60, 90, and 120 min, 2 mL samples were withdrawn from the dissolution medium and replaced with an equal volume of dissolution media to maintain a constant dissolution volume. Then, the samples were filtered through 0.45 µm PVDF membranes and diluted immediately with the mobile phase for HPLC analysis.

### 2.5. HPLC Analysis

A reverse phase HPLC system consisting of a Waters 2695 separation module, equipped with a Waters 2487 dual absorbance detector, was used for the quantitative analysis of CLT. The mobile phase consisted of a mixture of acetonitrile, methanol, and distilled water at a ratio of 7:3:10. The flow rate was maintained at 1 mL/min, and 20 µL of each sample was injected. The UV detector was set at 254 nm for analysis. The entire solution was filtered using a membrane filter (Millipore Corp., Bedford, MA, USA) and degassed before HPLC analysis.

### 2.6. Differential Scanning Calorimetry (DSC)

Thermal analysis of all the samples was performed using DSC (NETZSCH Gerätebau GmbH-DSC 200 F3 Maia^®^, Selb, Germany), at a heating rate of 10 K/min. Three milligrams of the sample was weighed, and dry nitrogen purge (50 mL/min) was employed in the process. Samples were scanned from 5 °C to 240 °C.

### 2.7. Fourier Transform Infrared (FTIR) Spectroscopy

Pure CLT, POX, SLS, PM, and SDs were characterized using FTIR spectrometry (FT-IR 4700 type A, Jasco, Easton, MD, USA). The samples were scanned in the wavelength range of 500–4000 cm^−1^, with a resolution of 2 cm^−1^.

### 2.8. Powder X-ray Diffraction (PXRD)

PXRD of pure CLT, POX, SLS, PM, and SDs was performed using a powder X-ray diffractometer (Smart Lab, Rigaku, Japan). The samples were scanned in a 2θ interval from 5° to 50°, with a scanning speed of 10°/min.

### 2.9. Field-Emission Scanning Electron Microscopy (FE-SEM)

The morphology of pure CLT, POX, SLS, PM, and SDs was examined using FE-SEM (JEOL, JMS 6700F, Tokyo, Japan), at an accelerating voltage of 5 kV. Samples were mounted onto a double-sided adhesive tape and sputter-coated with platinum, using an ion sputter.

### 2.10. Particle Size Distribution Analysis

The particle size distribution of pure CLT, POX, SLS, PM, and SDs (in solid-state powder) was analyzed using a laser diffraction particle size analyzer (HELOS/BF, Sympatec GmbH, Clausthal-Zellerfeld, Germany) after passing through a 40-mesh sieve. The particle diameters at 10% (D_10_), 50% (D_50_), and 90% (D_90_) were determined using the cumulative volumetric particle size distribution. SD powders were dispersed in 10 mL of water, and the particle size (in liquid-state) was evaluated by dynamic light scattering (ELS-8000, Otsuka Electronics Co. Ltd., Osaka, Japan).

### 2.11. Measurement of the Contact Angle

A 5% *w/v* solution of all samples was prepared in dimethyl sulfoxide. After filtration through a 0.2 µm PTFE membrane, 500 µL of the solution was spread uniformly onto a glass slide and dried at room temperature for 24 h to remove excess solvent. Then, 8 µL of distilled water was dropped on the slide glass. The contact angles were measured using a contact angle analyzer (Phoenix 150, SEO Contact Angle Analyzer, SEO Co., Suwon, Korea).

## 3. Results

### 3.1. Excipients Screening

The purpose of studying the solubility of drugs in aqueous solutions of different excipients was to screen the SD carriers and surfactants with optimal solubilization capacity. Table 2 shows the solubility of CLT in different media. The solubility of CLT was observed to be less than 9 µg/mL in aqueous solution without solubilizers. Among the solid carriers for preparing SD, POX greatly increased the solubility of CLT by two times as compared to the solubility of pure CLT in aqueous solution. Among all tested surfactants, SLS showed a remarkable increase in solubility of CLT, which was 130-fold higher than that of aqueous CLT alone, due to the formation of micellar SLS at the concentration above CMC (1% *w*/*v*). Therefore, POX and SLS were selected for further analysis.

### 3.2. Effect of SLS Micellization on Dissolution Profiles of CLT

Dissolution is the process whereby a solute dissolves in a solvent to form a solution as a function of time. Solubility is a thermodynamic process, showing the maximum concentration of a solute that can dissolve in a solvent at a given temperature. The solubility of the drug in the formulation was somewhat meaningful, but the dissolution performance of the drug in SD formulation, emphasizing the roles of micellar SLS, was more relevant for our goals. For many poorly water-soluble drugs, surfactants are mostly used in aqueous-based dissolution media to obtain a suitable sink condition. Among various surfactants, SLS was chosen because the solubility of CLT was increased tremendously (130-fold higher than aqueous CLT alone). SLS is also commonly used to develop a bio-relevant dissolution method. Herein, SLS at different concentrations (0.1%, 0.3%, and 0.5% *w*/*v*) was used in the surfactant-driven dissolution media. The dissolution profiles of pure CLT are presented in Figure S1. Without the presence of any surfactant in a pH 1.2 enzyme-free gastric fluid, the dissolution rate of CLT was lower than 3% after 2 h of testing, which was attributed to its extreme hydrophobicity. However, the presence of SLS in the dissolution medium significantly increased the dissolution of CLT to over 50% at 15 min. Interestingly, when the concentration of SLS in the medium was greater than the CMC of SLS, at 0.5%, the dissolution rate was observed to be more than 80%. Herein, the presence of SLS in an aqueous-based dissolution medium at the concentration above CMC could result in the formation of micelles for loading poorly water-soluble drugs and retarding the recrystallization process [32]. On the other hand, due to the toxicity concerns, it is challenging to design a solid dosage form containing SLS that could reach the concentration above CMC. Therefore, an attempt was made to elucidate the mechanism for improving dissolution of poorly soluble drugs after incorporating micellar SLS to the SD system.

To select an appropriate SD carrier, the effect of SD carrier types by solvent method on the dissolution profiles in enzyme-free gastric fluid (pH 1.2) is compared in Figure S2. A higher dissolution rate of CLT was observed from POX-based SD than from other carriers, and it was analogous to the solubility data (see Table 2) that the use of POX could achieve the highest solubility of CLT. Therefore, POX-based SDs were employed for further studies.

**Figure 1:**
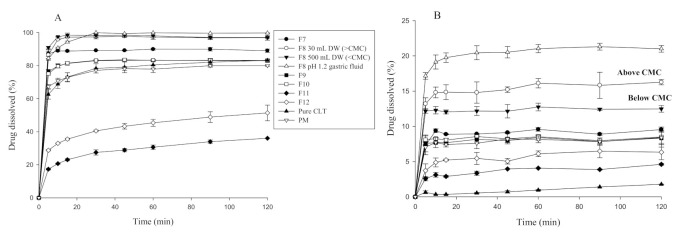
Dissolution profiles of cilostazol (CLT), physical mixture (PM), and solid dispersions (SDs) prepared by different methods in enzyme-free gastric fluid (pH 1.2) with (**A**) and without (**B**) 0.5% sodium lauryl sulfate (SLS) (*n* = 3).

SDs were then prepared by three different methods: fusion, solvent evaporation, and anti-solvent method. The effect of SLS on the dissolution rate of the CLT in the SDs system was elucidated in two different dissolution media (Figure 1). In enzyme-free gastric fluid (pH 1.2) containing 0.5% SLS (*w*/*v*) in Figure 1A, the percentage of dissolved drug from pure drug, PM, and SDs prepared by the fusion method stood at around 80% for 2 h without reaching 100%. Interestingly, the SDs prepared by the anti-solvent method showed the highest dissolution rate, reaching at 100% after 20 min. However, no significant difference between micellar SLS or SLS below CMC in SDs was observed due to the presence of SLS in dissolution media. In contrast, SDs prepared by the solvent method had a lower percentage of drug release than PM and pure CLT. This could be explained by the occurrence of drug recrystallization during the solvent evaporation which formed larger drug particles in SDs. This suggests that the pre-added incorporation of micellar SLS into POX-based SDs prepared by the anti-solvent method potentially played a vital role in facilitating drug solubilization and prevention of drug recrystallization during preparation.

Figure 1B shows the dissolution profiles of SDs in enzyme-free gastric fluid (pH 1.2) in the absence of SLS. This test was done to confirm the effect of SLS in SDs and to determine a pertinent method for the incorporation of SLS in SDs to achieve the highest dissolution rate. As expected, without SLS in the dissolution medium, the dissolution of the SDs containing SLS was still higher than that without SLS, similar to the dissolution phenomenon described earlier. The drug release of SDs prepared by the anti-solvent method was found to be higher than that of pure CLT and its PM. Furthermore, the addition of micellar SLS (30 mL in DW) above CMC in SDs synergistically increased the dissolution rate in gastric fluid (pH 1.2) without adding SLS as compared with SLS (500 mL in DW) below CMC (F8). There was a significant reduction of critical micellar concentration (CMC) of SLS (0.066 mg/mL) in enzyme-free pH 1.2 gastric fluid containing salt (Figure S3) as compared to in DW (2.34 mg/mL) [33]. Herein, the presence of salt in enzyme-free gastric fluid (pH 1.2) could reduce the CMC of SLS via a “salt-induced effect”, which was beneficial to exert more micellization of SLS in SDs and enhance the dissolution [34]. Collectively, when SLS was dissolved in an aqueous-based solution at the concentration above the CMC, it could form micelles as adjuvants to improve the solubilization of poorly water-soluble drugs and prevent the drug from aggregation. Simultaneously, this was reasonable for the improved dissolution rate of pure CLT in SLS-driven medium as compared with that in SLS-free medium. In addition, the anti-solvent method was better than the solvent/fusion method in promoting the effect of SLS-driven micellization in SDs for enhancing CLT dissolution.

### 3.3. DSC

**Figure 2:**
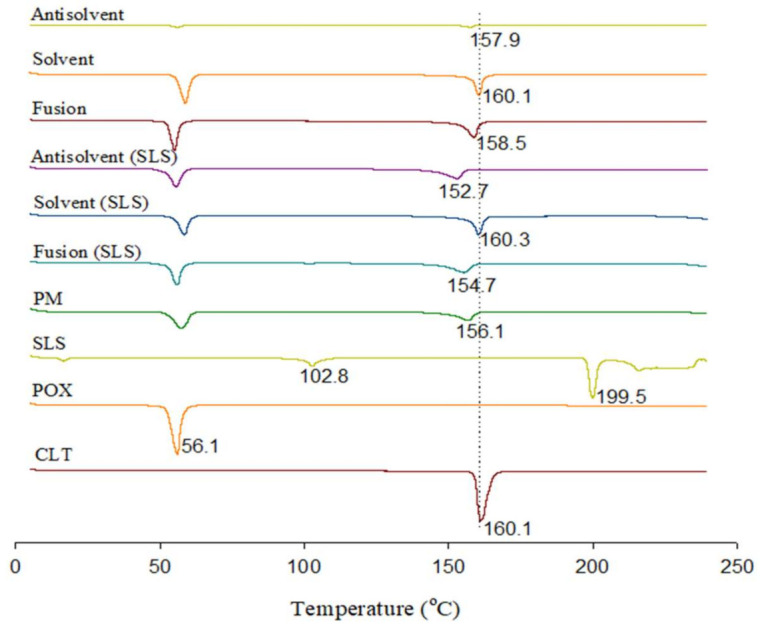
Differential scanning calorimetry thermograms of pure cilostazol (CLT), poloxamer, sodium lauryl sulfate (SLS), physical mixture (PM), and solid dispersions (SDs).

The DSC thermograms for each component and the CLT-SDs are presented in Figure 2. The endothermic peak of CLT was observed at the onset temperature of 160.1 °C, which corresponded to its melting point. Also, two characteristic peaks of SLS were described at 102.8 °C and 199.5 °C. The endothermic peaks of the drug in SDs, prepared by the solvent method with or without SLS, performed the same crystal peak as that in pure CLT, suggesting the recrystallization of the drug without polymorphic changes. Interestingly, as compared with other methods, the thermograms of SLS-loaded SDs prepared by the anti-solvent method showed a reduced peak intensity and shifted the crystal peak of CLT from 160.1 °C to 152.7 °C, at the lowest melting point, indicating the transformation of crystalline CLT into a partially amorphous state. Therefore, the addition of SLS to SDs by the anti-solvent method was beneficial in modulating the crystal habit of CLT to be more amorphous, resulting in further enhancement of drug dissolution.

### 3.4. FTIR

**Figure 3:**
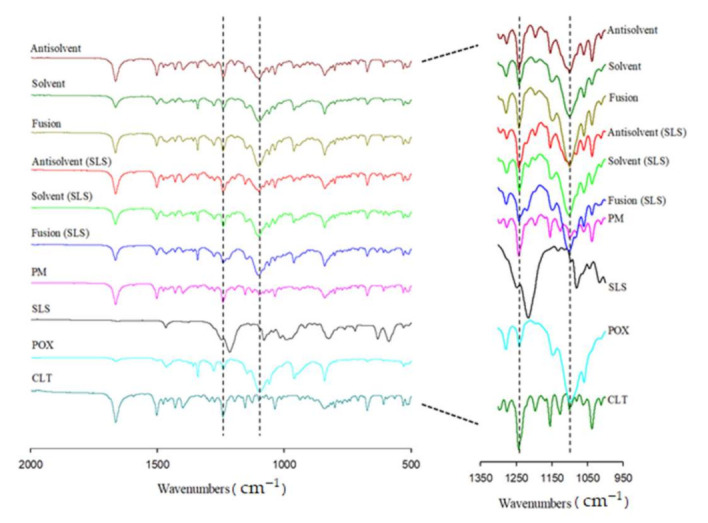
Fourier-transform infrared spectra of pure cilostazol (CLT), poloxamer, sodium lauryl sulfate (SLS), physical mixture (PM), and solid dispersions (SDs).

The FTIR spectra of pure CLT, POX, SLS, PM, and SDs are shown in Figure 3. Pure CLT showed a distinct N=N stretching peak of tetrazole at 1666 cm^−1^ and a C=C stretching band at 1504 cm^−1^ [35]. POX exhibited a characteristic peak at 1103 cm^−1^, ascribed to the stretching vibration of C-O-C [36]. Meanwhile, SLS exhibited a characteristic peak of –OSO_3_^-^ stretching at 1229 cm^−1^ [37]. Generally, the characteristic peaks of CLT in SDs, with or without SLS, appeared unchanged in SDs, indicating no interaction between CLT and carriers. At the same time, the peak of –OSO_3_^−^ stretching still remained in the spectra of SDs prepared by three methods. However, it was notable that the stretching vibration of C-O-C of POX in SDs prepared by the anti-solvent method with SLS was modified differently from pure POX and other SDs. This phenomenon indicates that there was an existence of hydrophobic interaction between the surfactant and the hydrophobic regions of the polymer chain via non-covalent bonding and adherence to the surface of nano-sized SLS micelles in the POX-based SDs matrix.

### 3.5. PXRD

**Figure 4:**
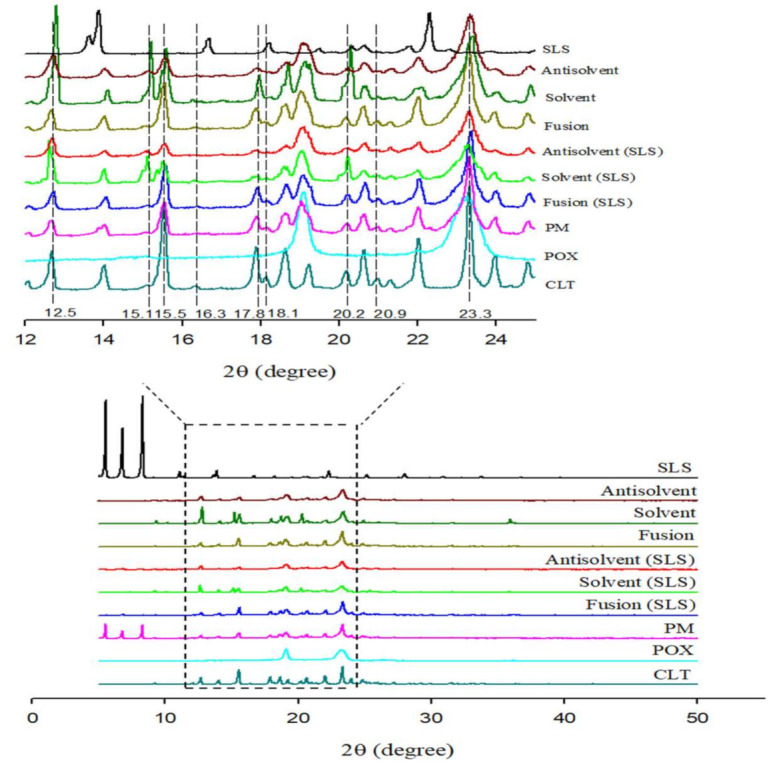
Powdered X-ray diffraction patterns of pure cilostazol (CLT), poloxamer, sodium lauryl sulfate (SLS), physical mixture (PM), and solid dispersions (SDs).

The PXRD patterns for each component and CLT-SDs are shown in Figure 4. For pure CLT, a series of distinct crystalline diffraction peaks were observed in the range of 12–30°, indicating that CLT was highly crystalline in nature. For PM, superposition of the diffraction peaks of the drug, POX, and SLS was observed. The crystalline diffraction peaks of CLT in SDs prepared by solvent methods with or without SLS, the appearance of two crystalline peaks at 15.1° and 20.2° provided evidence for the recrystallization of CLT after the preparation; thus, it was reasonable that the dissolution rate of CLT from SDs prepared by the solvent method was smallest as compared to other formulations, even untreated CLT. On the other hand, SDs prepared by the fusion and anti-solvent methods performed a reduction of crystalline intensity with two peaks at 12.5° and 17.8°. However, the most characteristic peaks of CLT in SDs prepared by the anti-solvent method were remarkably reduced, although some characteristic peaks of CLT still remained. The incorporation of SLS in SDs prepared by anti-solvent methods showed a significant reduction of numerous crystalline peaks of CLT positioned at 14°, 15.5°, 17.8°, 20.2°, and 23.3° and the disappearance of the peaks positioned at 16.3°, 18.1°, and 20.9°. Three characteristic peaks of SLS positioned at 5–8° also disappeared in SDs compared with the pattern of PM. Collectively, PXRD results revealed that the existence of SLS micelles in SDs prepared by the anti-solvent method led to the transformation of the crystalline drug to a more partially amorphous state, while SDs without SLS or SDs prepared by the solvent method were not sufficient to modulate the drug crystal habit.

### 3.6. FE-SEM

**Figure 5:**
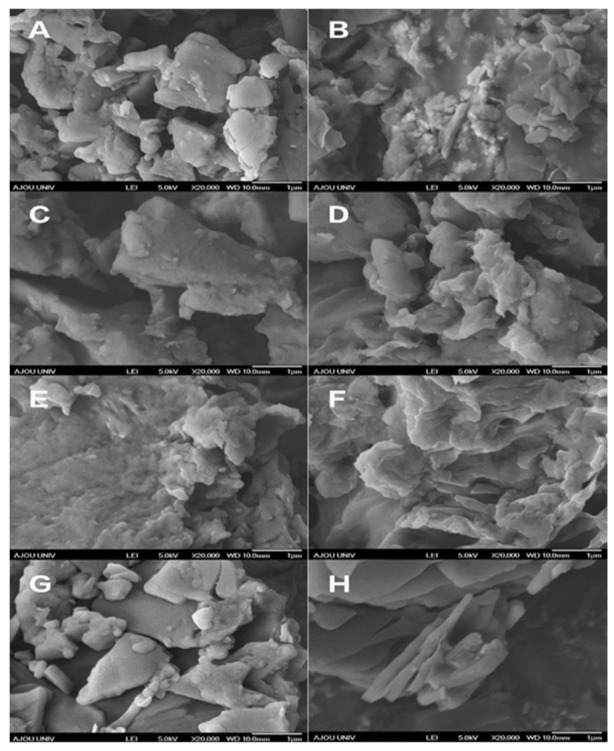
Field-emission scanning electron microscopy morphology of (**A**) pure cilostazol (CLT), (**B**) physical mixture (PM), (**C**) sodium lauryl sulfate (SLS)-added SDs (solid dispersions) prepared by the fusion method, (**D**) SLS-free SDs prepared by the fusion method, (**E**) SLS-added SDs prepared by the solvent method, (**F**) SLS-free SDs prepared by the solvent method, (**G**) SLS-added SDs prepared by the anti-solvent method, and (**H**) SLS-free SDs prepared by anti-solvent method.

The FE-SEM images of pure CLT, POX, SLS, PM, and SDs prepared by different methods are shown in Figure 5. Pure CLT presented a crystalline state, while the PM was composed of irregularly shaped particles of CLT, POX, and SLS. Although all SDs, with or without SLS, showed irregular shapes, indicating the amorphous state of CLT, SD prepared by the anti-solvent method with SLS showed a smaller particle size distribution and smoother surface than that in the same formulation without SLS. These profiles evidenced that SDs prepared by the anti-solvent method with SLS possessed an improvement of drug solubilization as compared to pure CLT and other SDs.

### 3.7. Solid Particle Size Distribution

As shown in Table 3, pure CLT had the smallest solid particle size, but the in vitro dissolution rate was extremely low because of its poor water solubility. Although the particle size of the PM was smaller than that of the SDs, PXRD results indicated that the drug still existed in a crystalline state because of the absence of any interaction between the drug and the excipients, and the release rate was less than that of the SDs. A comparison of the particle size distribution of the SDs prepared by the three methods clearly showed that the particle size of SDs prepared by the anti-solvent method was reduced significantly. Furthermore, the adding of micellar SLS in SDs, especially for SDs prepared by anti-solvent methods, obtained the smallest solid particle size distribution via adherence of dissolved CLT to the surface of nano-sized SLS micelles into the POX-based SD matrix, preventing the drug from aggregation during preparation by the anti-solvent method.

### 3.8. Effect of SLS Micellization in SD Systems

The particles size before and after dispersion in media should be used for the interpretation of roles of micellar SLS and dissolution behaviors of CLT in SDs. As shown in Table 4 (see also Figure 1B for dissolution), the inclusion of SLS above CMC in the SDs prepared by the anti-solvent method had a significant effect on the particle size distribution. Incorporation of SLS in SDs could avoid the aggregation of drug particles, owing to the adherence of dissolved CLT molecules to the surface of SLS micelles during the Ostwald ripening process, preventing the drug from recrystallization, from which the particle size of CLT prepared by the anti-solvent method before drying and after redispersing in water was not significantly different [38]. Furthermore, the particle size distribution (D_50_) of the dried SLS-loaded SDs before and after dissolution was smaller than that of the SDs without SLS. Taken together, the micellization of SLS incorporated in SDs played a vital role in preventing CLT aggregation during SD preparation and in the aqueous dissolution process.

### 3.9. Analysis of the Wetting Properties

**Figure 6:**
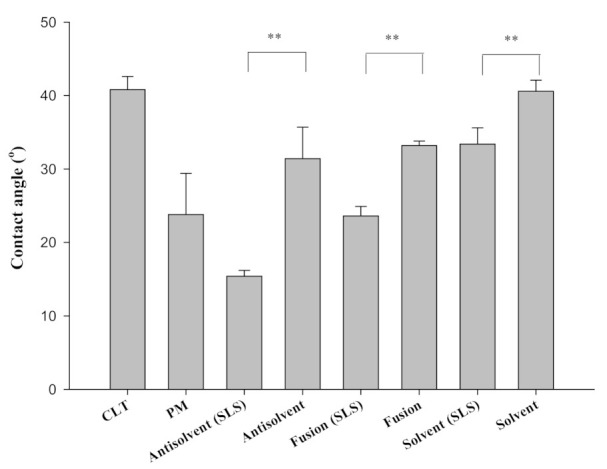
Contact angle of pure cilostazol (CLT), physical mixture (PM), and SDs (solid dispersions). (**: *p* < 0.01).

Contact angle is also one of key factors to expect wettability of solid dosage forms. Figure 6 shows the contact angle images of SDs prepared by three different methods. As expected, the contact angle of SDs containing SLS was clearly smaller than that of SDs without SLS, thus suggesting that SLS improved the wettability and hydrophilicity of the drug. The contact angle as an index of wetting was varied by the three SD preparation methods. Interestingly, SDs prepared by the anti-solvent method with SLS exhibited the lowest contact angle measurement, indicating the highest wettability and its superiority in reducing the drug crystallinity and improving the dissolution of CLT.

### 3.10. Commentary for Mechanistic Roles of Micellar SLS

**Figure 7:**
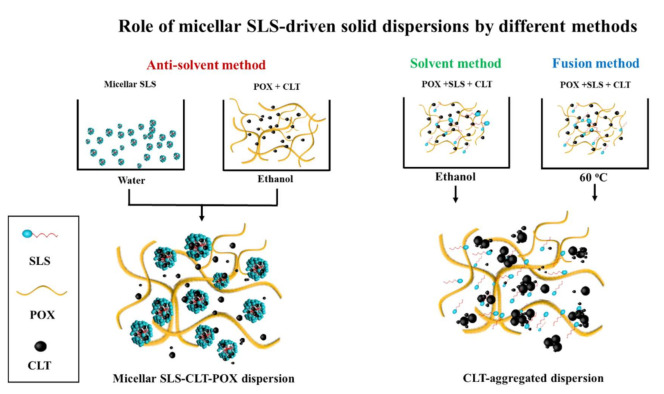
Schematic diagram of the role of micellar SLS-driven solid dispersion by different methods, showing dissolution enhancement of the anti-solvent method.

The mechanistic diagram of the role of micellar SLS-driven solid dispersion by different methods is shown in Figure 7. When the micellar SLS was prepared by decreasing solvent volume and pre-added into the SD system during the anti-solvent methods, the dissolved CLT molecules adhered to the surface of nano-sized SLS micelles in the POX network via the Ostwald ripening process, resulting in the reduced drug crystallinity, the lowest contact angle, the smaller particle size, and the non-covalent hydrophobic interaction between the surfactant and the hydrophobic regions of polymer, as compared with fusion and solvent evaporation methods. In contrast, the fusion and solvent evaporation methods were not effective at the same concentration of SLS, inducing aggregation and precipitation in the SD preparation process. The initial presence of SLS micelles by reducing solvent volume could increase the possibility of adherence of the dissolved CLT to SLS micelles in the POX matrix network, preventing drug recrystallization and enhancing the wettability and dissolution rate of extremely water-insoluble CLT from SDs.

## 4. Conclusions

In this study, we investigated the role of SLS in SDs prepared by three different methods: fusion, solvent, and anti-solvent methods. The solubility test indicated that SLS was the most powerful surfactant in enhancing the solubility of CLT as compared to other carriers and surfactants. Furthermore, the presence of SLS in dissolution media to obtain sink conditions significantly enhanced the dissolution rate of pure CLT. Interestingly, our findings revealed that the anti-solvent method was an effective method for improving the dissolution rate of CLT in SDs in which the initial micellization of SLS in the aqueous phase at the concentration above CMC was carried out to prevent CLT from recrystallization and precipitation in dissolution media. Furthermore, PXRD and FTIR results revealed the molecular interactions between SLS micelles and POX-based CLT SD, resulting in reduced drug crystallinity in SD prepared by the anti-solvent method. Collectively, our findings suggest that the pre-added incorporation of micellar SLS into POX-based SDs using the anti-solvent method could provide a way of a solubilization mechanism to enhance the dissolution rate of extremely water-insoluble CLT.

## Figures and Tables

**Table 1:** Formulation compositions (mg) and preparation methods of CLT-SDs.

Code	CLT	POX	PVP	Soluplus	TPGS	PEG6000	SLS	Method
F1	50	150	—	—	—	—	—	Solvent
F2	50	—	150	—	—	—	—	Solvent
F3	50	—	—	150	—	—	—	Solvent
F4	50	—	—	—	150	—	—	Solvent
F5	50	—	—	—	—	150	—	Solvent
F6	50	50	—	—	—	—	10	Physical mixture
F7	50	50	—	—	—	—	—	Antisolvent
F8	50	50	—	—	—	—	10	Antisolvent
F9	50	50	—	—	—	—	—	Fusion
F10	50	50	—	—	—	—	10	Fusion
F11	50	50	—	—	—	—	—	Solvent
F12	50	50	—	—	—	—	10	Solvent

Abbreviations: CLT, cilostazol; POX, poloxamer; PVP, polyvinylpyrrolidone; TPGS, D-α-tocopherol polyethylene glycol 1000 succinate; PEG6000, polyethylene glycol 6000; SLS, sodium lauryl sulfate.

**Table 2:** Solubility of CLT in distilled water, different pH buffers, and 1% *w/v* aqueous solutions of various excipients.

Media	Solubility (µg/mL)	Role	Physical State
Deionized water	8.03 ± 1.05	Medium	Liquid
Gastric fluid (pH 1.2)	8.41 ± 0.96	Medium	Liquid
Intestinal fluid (pH 6.8)	7.13 ± 0.14	Medium	Liquid
TPGS	6.79 ± 0.71	Carrier	Semisolid
Soluplus	14.68 ± 1.46	Carrier	Solid
PVPk30	7.77 ± 0.19	Carrier	Solid
PEG6000	8.05 ± 0.41	Carrier	Solid
POX	17.11 ± 0.87	Carrier	Solid
PEO	12.44 ± 3.85	Carrier	Solid
Primojel	6.90 ± 0.23	Carrier	Solid
HPMC 6cps	11.69 ± 0.29	Carrier	Solid
Gelucire 44/14	25.37 ± 4.06	Surfactant	Semisolid
Span 20	11.55 ± 0.54	Surfactant	Liquid
Span 80	8.54 ± 0.50	Surfactant	Liquid
Brij 35	45.32 ± 2.96	Surfactant	Semisolid
Brij 56	33.99 ± 3.13	Surfactant	Semisolid
Brij 92	9.24 ± 0.56	Surfactant	Semisolid
Brij 97	46.39 ± 3.98	Surfactant	Semisolid
Brij 98	52.88 ± 2.08	Surfactant	Semisolid
Ariacel 83	9.17 ± 0.16	Surfactant	Semisolid
Tween 80	39.07 ± 0.86	Surfactant	Liquid
SLS	1170.54 ± 1.05	Surfactant	Solid

Abbreviations: CLT, cilostazol; POX, poloxamer; PVP, polyvinylpyrrolidone; TPGS, D-α-tocopherol polyethylene glycol 1000 succinate; PEG6000, polyethylene glycol 6000; SLS, sodium lauryl sulfate, PEO, poly(ethylene oxide); HPMC 6cps, hydroxypropyl methylcellulose 6cps.

**Table 3:** Particle size distribution of PM and SDs according to formulation compositions and preparation methods.

Code	CLT (mg)	POX (mg)	SLS (mg)	D_10_ (µm)	D_50_ (µm)	D_90_ (µm)	Method
CLT	50	—	—	1.76	7.44	23.23	—
POX	—	50	—	28.48	131.94	379.57	—
SLS	—	—	10	5.32	65.37	254.23	—
F6	50	50	10	3.24	34.06	196.78	PM
F7	50	50	—	4.29	40.82	303.15	Antisolvent
F8	50	50	10	3.62	27.4	261.28	Antisolvent
F9	50	50	—	34.75	227.98	394.26	Fusion
F10	50	50	10	9.22	115.84	367.9	Fusion
F11	50	50	—	12.77	125.71	376.99	Solvent
F12	50	50	10	14.14	123.49	352.17	Solvent

**Table 4:** The effect of SLS on the particle size (D_50_, µm) of SDs prepared by the anti-solvent method with different processing periods.

Sample	SLS		Particle Size (µm)	
		Before Drying	After Drying	Redispersion ^a^
F7	No	1.17 ± 0.06	37.44 ± 4.78	3.51 ± 0.29
F8 30mL DW ^b^	Yes	2.06 ± 0.27	69.09 ± 1.06	2.36 ± 0.3
F8 30mL GF (pH 1.2) ^b^	Yes	3.32 ± 0.03	93.38 ± 3.05	3.73 ± 0.19

^a^ Re-dispersed dried SDs for 2 h in SLS-free gastric fluid (pH 1.2). The particle size was unable to be determined in SLS-added gastric fluid (pH 1.2). ^b^ SLS concentration above critical micellar concentration.

## Data Availability

No new data were created or analyzed in this study. Data sharing is not applicable to this article.

## Supplementary Materials

The following are available online at https://www.mdpi.com/article/10.3390/pharmaceutics13050662/s1, Figure S1: Dissolution profiles of pure CLT in enzyme-free gastric fluid (pH 1.2) with/without surfactant (*n* = 3); Figure S2: Effect of SD carrier types by solvent method on the dissolution profiles in enzyme-free gastric fluid (pH 1.2) without SLS (*n* = 3); Figure S3: Profile of critical micelle concentration determination of SLS in enzyme-free gastric fluid (pH 1.2).
