# Supplementary material for: Role of Surfactant Micellization for Enhanced Dissolution of Poorly Water-Soluble Cilostazol Using Poloxamer 407-Based Solid Dispersion via the Anti-Solvent Method
Source: Pharmaceutics. 2021 May 5;13(5):662. doi: 10.3390/pharmaceutics13050662 (PMC8148127; doi:10.3390/pharmaceutics13050662)
Supplement: Supplementary file 1 [file pharmaceutics-13-00662-s001.zip › pharmaceutics-1168372-supplementary.pdf]

pharmaceutics

Supplementary Materials: Role of Surfactant Micellization for Enhanced Dissolution of Poorly Water-Soluble Cilostazol Using Poloxamer 407-Based Solid Dispersion via the Anti-Solvent Method

Gang Jin, Hai V. Ngo, Jing-Hao Cui, Jie Wang, Chulhun Park and Beom-Jin Lee

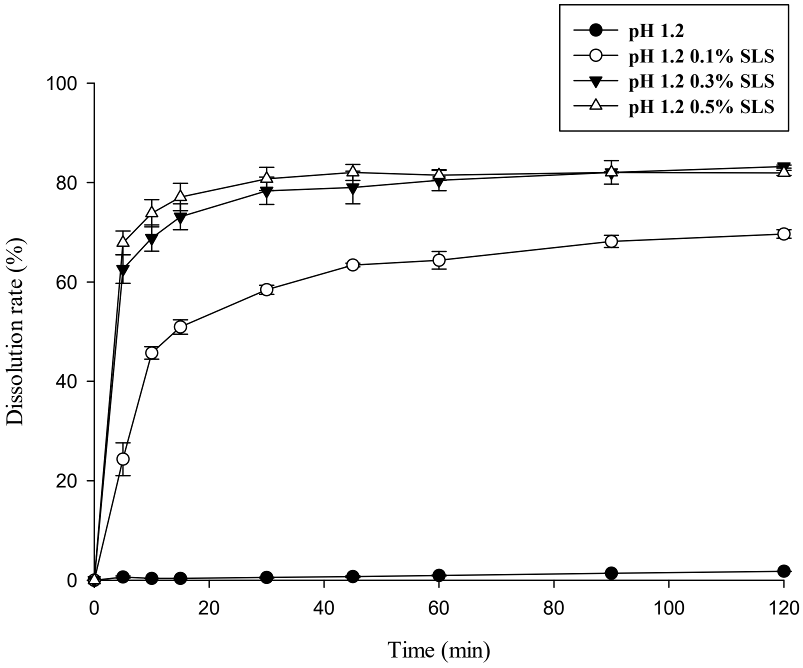

Figure S1. Dissolution profiles of pure CLT in pH 1.2 with/without surfactant (*n* =3).

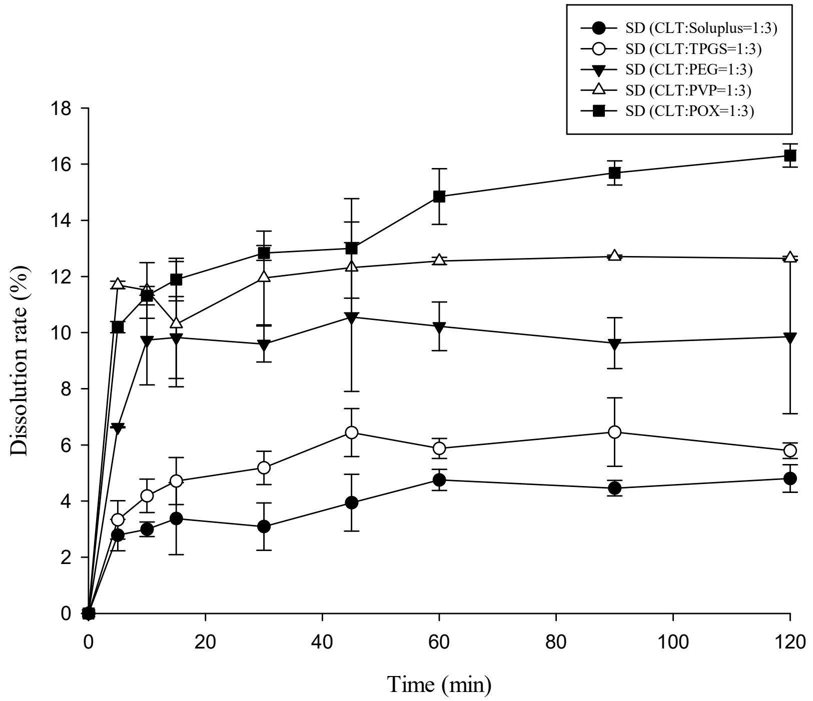

Figure S2. Effect of SD carrier types by solvent method on the dissolution profiles in SLS-free buffer pH 1.2 gastric fluid (*n* =3).

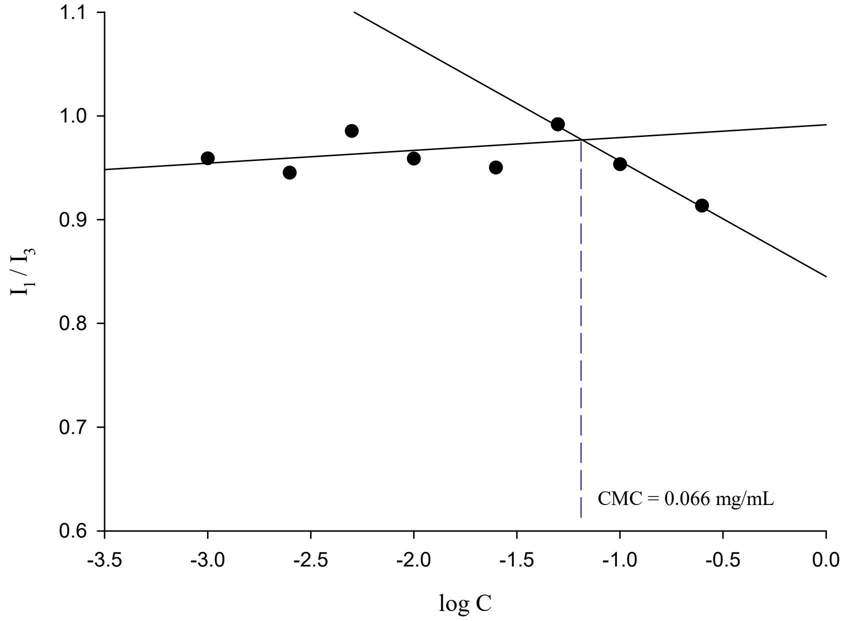

Figure S3. Profile of critical micelle concentration determination of SLS in buffer pH 1.2.
